# Lactate-Mediated Epigenetic and Immunometabolic Reprogramming in Glioma: An Emerging Axis Linking Metabolism to Tumor Progression

**DOI:** 10.3390/biomedicines13123041

**Published:** 2025-12-11

**Authors:** Xinyi Xie, Wenjing Zhou, Yin Ku, Shasha Li, Yunhao Yang, Xiaohu Hao, Yaohui Chen

**Affiliations:** Department of Thoracic Surgery and Institute of Thoracic Oncology, Frontiers Science Center for Disease-Related Molecular Network, West China Hospital, Sichuan University, Chengdu 610097, China

**Keywords:** gliomas, lactate metabolism, lactylation, metabolic reprogramming, immune microenvironment

## Abstract

**Background**: Among primary malignant brain tumors in adults, glioblastoma is the most common and the most aggressive, characterized by profound metabolic reprogramming. This metabolic shift is essential for sustaining relentless proliferation and adapting to the challenging tumor microenvironment (TME). Central to this adaptation in glioma is the Warburg effect, which leads to excessive lactate production and accumulation, even in the presence of oxygen. This metabolic divergence significantly impacts the tumor immune microenvironment, promoting the recruitment of immunosuppressive cells and weakening the anti-tumor immune response. **Core Content:** This review provides a comprehensive analysis of the multifaceted roles of lactate in IDHwt glioma pathogenesis. It explores how lactate serves as a critical nexus connecting aberrant metabolism, epigenetic reprogramming (notably via histone lactylation), and immune evasion. The review delves into the molecular mechanisms by which lactate, particularly through the post-translational modification known as lactylation, directly modulates the epigenetic landscape to promote oncogene expression. Furthermore, it examines lactate’s role in acidifying the TME, promoting the immunosuppressive M2 polarization of glioma-associated macrophages (GAMs), and inhibiting the cytotoxic activity of T lymphocytes. **Conclusions**: This “lactate-centric” framework provides a unifying model that links metabolic dysregulation directly to malignant progression and therapeutic resistance (e.g., to TMZ). By elucidating this metabolic–epigenetic–immune axis, the review highlights a critical dependency that fuels glioma aggression. Finally, it discusses emerging therapeutic strategies aimed at targeting lactate production (LDHAi), transport (MCTi), and downstream epigenetic signaling (HDACi/p300i), offering novel avenues for integrative immunometabolic therapy.

## 1. Introduction

Gliomas represent a category of malignant primary tumors originating in the adult central nervous system (CNS) and are associated with an extremely poor prognosis, with glioblastoma (GBM) exhibiting the highest degree of malignancy. The 2021 WHO classification shifts diagnosis from morphology to integrated molecular–pathologic criteria, emphasizing biomarkers like IDH mutation and 1p/19q codeletion. Major diffuse glioma entities are IDH-mutant astrocytoma, IDH-mutant/1p/19q-codeleted oligodendroglioma, and IDH-wildtype glioblastoma (GBM)—the latter being the most common and aggressive. Adult diffuse gliomas are predominantly composed of IDH-wildtype glioblastomas (~70–75%), which are associated with a median overall survival (OS) of approximately 14–18 months. In contrast, IDH-mutant astrocytomas (~10–15%) and oligodendrogliomas harboring 1p/19q co-deletion (~4–5%) exhibit significantly better outcomes, with a median OS ranging from 8 to 12 years or longer. Epidemiological data indicate that gliomas constitute over 20% of all primary brain and CNS tumors. Glioblastoma is the most common malignant subtype, accounting for 14.0% of all primary brain and CNS tumors and 51.5% of all malignant ones. This disease is characterized by an extremely poor prognosis, with a five-year survival rate of only 7.1% [1]. Among primary malignant brain tumors in adults, IDH-wildtype glioblastoma is the most common and the most aggressive, with the poorest prognosis. Despite continuous advancements in conventional treatment modalities, including surgery, radiotherapy, and chemotherapy, therapeutic efficacy remains minimal. This reality underscores the urgent need to move beyond the traditional genetic perspective and to explore the deeper, more plastic regulatory networks by which malignant tumor progression is driven.

In addition to genetic and epigenetic alterations, metabolic reprogramming has been recognized as a core pathological feature of glioma. One hallmark alteration is the preferential utilization of aerobic glycolysis for energy production, even under oxygen-sufficient (normoxic) conditions—a phenomenon known as the Warburg effect [2]. This metabolic shift ultimately results in the substantial accumulation of its primary metabolic end product, lactate, within the tumor microenvironment (TME) [3,4]. Lactate is transported between tumor cells and stromal cells via monocarboxylate transporters (MCTs), establishing a concentration gradient-driven “lactate shuttle”. Within this shuttle, MCT1 primarily mediates lactate uptake, while MCT4 is predominantly responsible for its efflux, thereby promoting metabolic symbiosis and an acidic ecosystem within the TME. This phenomenon has been documented in multiple cancer types, as supported by both review literature and experimental evidence in glioma [5,6,7].

Historically, lactate was once considered a “waste product” of cellular metabolism; however, research over the past decade has fundamentally revised this traditional view, by revealing its pleiotropic roles as both a key signaling molecule and an epigenetic regulator. This paradigm shift in understanding was precipitated by a groundbreaking discovery: the identification of histone lactylation (Kla), a novel post-translational modification (PTM) [8]. In that research, it was demonstrated that endogenous lactate can directly serve as a substrate to modify lysine residues on histones, consequently remodeling chromatin structure and modulating gene transcription. This discovery provides a direct molecular bridge linking cellular metabolic status to gene expression regulation. This mechanism is of particular importance in the pathological context of glioma. Multiple studies have indicated that lactate-induced epigenetic reprogramming is closely associated with both the maintenance of glioma stem cells (GSC) and malignant tumor progression, constituting a key “lactate-epigenetics-tumor progression” pathogenic axis [9,10,11].

The effects of this lactate-driven epigenetic reprogramming extend far beyond the tumor cells themselves, profoundly reshaping the tumor immune microenvironment. High concentrations of lactate, and the accompanying microenvironment acidification, are capable of systemically suppressing anti-tumor immunity by modulating the “immuno-epigenome” of resident immune cells. Specifically, high lactate and acidification inhibit the metabolism and gene expression of anti-tumor effector cells, such as CD8^+^ T cells and dendritic cells [12,13]. These conditions also induce myeloid cells, such as TAMs, MDSCs, to acquire an immunosuppressive phenotype, a fate decision that is subsequently consolidated through epigenetic modifications [9,14]. These effects have been progressively validated and summarized in studies concerning multiple cancer types and brain tumors [15]. Notably, the regulation of immune cells by lactate appears to be context-dependent: under pH-controlled conditions, the lactate molecule itself may enhance the stemness and persistence of CD8^+^ T cells via mechanisms including the inhibition of histone deacetylases (HDACs) [16]. This complexity suggests that the lactate molecule and its resultant acidic environment may play different, or even opposing, roles in immune regulation. an understanding of this duality is therefore crucial for the rational design of immunotherapy strategies.

In summary, lactate is no longer viewed merely as a simple glycolytic product; it is now recognized as a key hub connecting the three core biological processes of metabolic reprogramming, epigenetic regulation, and immune remodeling. On the one hand, metabolic reprogramming promotes lactate enrichment, which subsequently remodels chromatin and transcriptional networks via pathways such as Kla. On the other hand, lactate modulates the “immuno-epigenetic” program of immune cells, which in turn impacts tumor metabolism and progression, thereby establishing a positive feedback loop. It must be emphasized that this lactate-centric regulatory axis exhibits significant heterogeneity across different molecular subtypes of glioma. In isocitrate dehydrogenase (IDH) wild-type (IDHwt) gliomas, particularly GBM, the Warburg effect is more pronounced and lactate levels are higher, rendering this regulatory axis a core pathogenic mechanism. In contrast, IDH-mutant (IDHmut) gliomas are characterized by lower lactate levels, and their epigenetic landscape is driven primarily by the oncometabolite D-2-hydroxyglutarate (D-2HG). Accumulating evidence from both clinical and preclinical studies has demonstrated that intratumoral lactate levels are significantly higher in IDH-wt gliomas compared to IDH-mut tumors. Notably, in a study utilizing 31P/1H-MRS, Wenger et al. reported a marked disparity in in vivo lactate concentrations between these subgroups, with mean levels of 5.4 ± 4.1 mmol/L in IDH-mut gliomas versus 11.7 ± 4.3 mmol/L in IDH-wt counterparts [17].

Therefore, this review will focus on IDHwt glioma, systematically elucidating: (i) how metabolic alterations in glioma promote lactate accumulation and drive epigenetic reprogramming, and (ii) how lactate mediates immuno-epigenetic regulation of various immune cells within the TME. Building upon this foundation, we will further integrate the synergistic effects of these two processes in tumor progression and explore therapeutic strategies targeting key nodes along this axis. This discussion aims to provide a theoretical framework and delineate future directions for the development of precise immune–metabolic–epigenetic combination therapies for glioma. To systematically construct this theoretical framework, it is first necessary to analyze in-depth the metabolic reprogramming mechanisms responsible for lactate accumulation in glioma, as well as the molecular basis by which epigenetic changes are driven.

## 2. Metabolic Reprogramming and Lactate-Driven Epigenetic Alterations in Glioma

**Figure 1:**
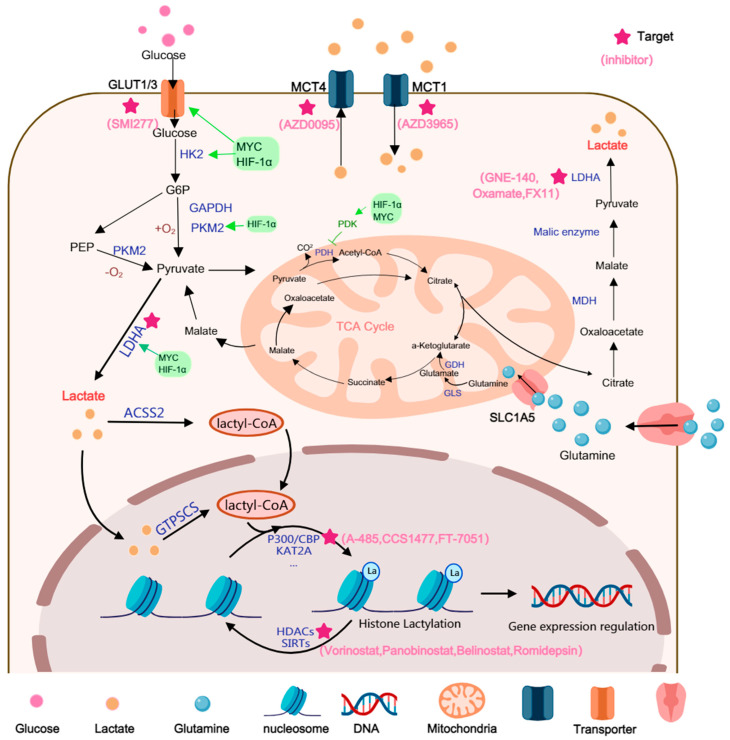
Lactate can be generated through multiple pathways. Lactate is primarily generated via the aerobic glycolysis pathway and glutaminolysis (the glutamine pathway). In GBM, glucose metabolic re-programming enhances glucose uptake, which is mediated by the glucose transporters GLUT1/3. Enzymes such as HK2, PKM2, and LDHA are upregulated, often under the modulation of multiple signaling factors, to accelerate lactate generation. Glutamine is transported into the mitochondria, where it is converted to α-ketoglutarate by GLS. This α-ketoglutarate subsequently enters the TCA cycle to generate malate, which is then exported from the mitochondria to be converted into lactate. Lactate is converted into lactyl-CoA both within and outside the nucleus. Subsequently, the lactyl group is transferred from lactyl-CoA onto histone residues by lactylation ‘writers’, ultimately modulating gene regulation in cancer cells.

Glioma cells exhibit a glycolytic phenotype with upregulated LDHA, PKM2, and MCTs, resulting in elevated intracellular and extracellular lactate levels. This accumulation profoundly affects the epigenetic landscape by serving as a substrate for histone lactylation and by inhibiting histone deacetylases (HDACs). p300-dependent histone lactylation has been shown to activate genes involved in metabolic adaptation, angiogenesis, and cellular stress responses. These lactate-induced epigenetic modifications contribute to transcriptional reprogramming that reinforces the glycolytic state, enhances stemness, and promotes therapy resistance in glioma (Figure 1). 

### 2.1. Glycolytic Shift and Lactate Accumulation in Glioma

Since the discovery of lactate in 1780, its metabolic role and significance in oncology have been progressively elucidated. Advances in isotope tracing, single-cell sequencing, and probe-based metabolic imaging have enabled the biological properties and functions of lactate and lactylation to be extensively explored [8]. A classic form of metabolic reprogramming in tumors is the Warburg effect, which is closely associated with lactate production [4,18]. It is generally assumed that, similar to other malignancies, GBM consumes large quantities of glucose and metabolizes it into lactate via pyruvate.

The pronounced glycolytic phenotype in glioma is not a form of passive cellular adaptation; rather, it is actively driven by a core oncogenic transcriptional program. In the glioma microenvironment, hypoxic or pseudohypoxic conditions are frequently observed within the tumor core, leading to the stabilization and activation of key transcription factors, including Hypoxia-inducible factor-1α (HIF-1α) and MYC [19,20].

These master transcriptional regulators act synergistically to systematically upregulate the expression of a suite of genes across the entire glycolytic pathway, which compels a shift in the cellular metabolic phenotype towards aerobic glycolysis terminating in lactate production. HIF-1α is known to directly transcriptionally activate glycolysis-related genes, including GLUT1/GLUT3, HK1/2, PGK1, PKM2, LDHA, and PDK1 [21]. The action of PDK1 is particularly notable, as it inhibits pyruvate entry into the mitochondria, which further channels metabolism towards the lactate pathway. Additionally, c-Myc is another oncogenic transcription factor that targets glycolytic enzymes such as PDK1, LDHA, HK2, and TFRC. The resulting overexpression of these enzymes has been shown to promote GBM cell proliferation, therapeutic resistance, and intracranial growth [22].

In glioma, hypoxic and pseudohypoxic signaling compels tumor cells to maintain a preference for glycolysis even under aerobic conditions, ultimately driving lactate production. This process is initiated by increased glucose uptake. GLUT1/GLUT3, the predominant glucose transporters in the human brain, enable glioma cells to efficiently sequester glucose from the microenvironment [23]. High GLUT3 expression is associated with tumor stemness, survival under nutrient-deprived conditions, and invasiveness, while GLUT1 upregulation is highly coupled to HIF-1α activation [24,25].

Upon cellular entry, glucose is rapidly catalyzed by a series of key rate-limiting enzymes that are transcriptionally regulated by HIF-1α/MYC. Specifically, Hexokinase 2 (HK2) catalyzes the first phosphorylation step, while the M2 isoform of Pyruvate kinase (PKM2) catalyzes the final step of glycolysis, ultimately promoting the conversion of glucose to pyruvate [26,27]. Notably, PKM2 functions not only as a metabolic enzyme; its dimeric form can also enter the nucleus and act as a transcriptional co-activator. In this role, it synergizes with HIF-1α and other factors to further amplify the expression of glycolysis-related genes, establishing a positive feedback loop.

At the terminal step of glycolysis, the conversion of pyruvate to lactate is catalyzed by LDHA, a process that also regenerates NAD^+^. This enzyme is indispensable for maintaining a high glycolytic flux. Multi-omics analyses in GBM patient cohorts have revealed that LDHA levels are elevated in GBM compared to low-grade glioma (LGG), and are correlated with a poor prognosis [28]. Furthermore, in mouse GBM models (GL261/CT2A), LDHA silencing has been shown to inhibit tumor growth and prolong survival. It also impacts tumor-macrophage interactions and the immune microenvironment, indicating that it functions as both a metabolic node and an immune-regulatory pivot [9]

In addition to glycolysis, lactate can also be generated via glutamine metabolism. Glutamine metabolism is initiated by the cellular uptake of glutamine, a process mediated by the Alanine-Serine-Cysteine Transporter 2 (ASCT2) [29]. Once transported into the cell, glutamine is shuttled into the mitochondria and is subsequently converted to glutamate, and then to α-ketoglutarate (α-KG), by the enzyme glutaminase (GLS). This α-KG can then enter the tricarboxylic acid (TCA) cycle. Ultimately, intermediates from this pathway are transported to the cytoplasm and converted to NADPH and pyruvate. Furthermore, alanine transaminase (ALT) catalyzes the reversible transamination between alanine and α-KG, which generates pyruvate and glutamate as part of cellular glutamine catabolism [30].

Lactate transport across cellular membranes is mediated by reversible monocarboxylate transporters (MCTs) [31]. The MCT family facilitates lactate exchange across the plasma membrane via H+/lactate symport. Lactate efflux through MCTs removes protons, thereby aiding in the maintenance of cytoplasmic pH balance while concurrently inducing acidification of the extracellular environment. The distribution of lactate flux across the cell membrane is governed by these transporters. Specifically, MCT4 is primarily responsible for efflux, while MCT1 mediates uptake, collectively establishing a “lactate shuttle” between tumor and stromal cells [32]. Both histological and functional studies involving multiple cohorts have demonstrated that MCT1 (SLC16A1) and MCT4 (SLC16A3) are overexpressed in GBM and are associated with malignant phenotypes. For example, in one study integrating human GBM cell lines, zebrafish xenografts, and patient biopsies, it was found that exogenous lactate treatment significantly promoted GBM cell proliferation/migration while concurrently upregulating MCT1 and the lactate receptor HCAR1 [33]. Previous IHC studies have also reported the overexpression of MCT1, MCT4, and their chaperone protein CD147 in GBM tissue. Expression levels were found to be significantly elevated in comparison to non-tumor brain tissue, with localization observed to be predominantly at the membrane, a finding consistent with their transmembrane transport function [34].

However, the metabolic landscape of glioma is not monolithic; it is profoundly influenced by the tumor’s IDH gene mutation status. Mutations in the IDH1 genes confer a neomorphic catalytic activity upon the enzyme, enabling the reduction of α-ketoglutarate (α-KG) to D-2HG [35,36]. D-2HG, which functions as an “oncometabolite,” competitively inhibits a range of α-KG-dependent dioxygenases (including TET DNA demethylases and JmjC histone demethylases) [37,38]. This inhibitory action leads to widespread DNA and histone hypermethylation, resulting in the formation of what is known as the “Glioma CpG Island Methylator Phenotype” (G-CIMP) [39,40].

At the metabolic level, glycolysis and lactate flux in IDHmut tumors are typically lower than those observed in IDHwt counterparts. Multi-cohort imaging studies have indicated that IDHmut gliomas possess lower intratumoral lactate levels, which are accompanied by a higher intracellular pH [17]. Intratumoral lactate concentrations differ significantly between patients with IDH-mut and IDH-wt gliomas. Specifically, the mean lactate level in IDH-mutant tumors is 5.4 ± 4.1 mmol/L, compared to approximately 11.7 ± 4.3 mmol/L in IDH-wildtype tumors. Analyses of biopsy data from The Cancer Genome Atlas (TCGA) low-grade glioma cohort revealed that MCT1/MCT4 are downregulated in IDHmut tumors, suggesting a reduction in lactate transmembrane flux and a diminished “lactate shuttle” [41]. In stark contrast, IDHwt gliomas, particularly primary GBM, exhibit a more pronounced Warburg effect and greater lactate efflux, which results in a more acidic microenvironment characterized by higher lactate concentrations. Therefore, this lactate-enriched microenvironment, which is driven by high-glycolytic flux, represents a defining feature of IDHwt glioma. This environment provides both an abundant substrate and a strong driving force for the lactate-driven epigenetic and immune-regulatory events that will be discussed in subsequent sections [42].

### 2.2. Lactate as an Epigenetic Substrate in Glioma

Epigenetic regulation is defined as the modulation of gene expression that occurs without altering the underlying DNA sequence; these regulatory mechanisms include DNA methylation, histone modifications, chromatin remodeling, and the action of non-coding RNAs. Tumor initiation and progression are accompanied by widespread epigenetic dysregulation. This dysregulation not only drives the activation of oncogenes and the silencing of tumor suppressor genes but also profoundly influences tumor immunogenicity and the immunosuppressive state of the tumor microenvironment (TME).

As previously discussed, the substantial accumulation of lactate in IDHwt glioma establishes a direct bridge between cellular metabolic status and chromatin biology. The key mechanism is the participation of lactate as a substrate in a novel post-translational modification: histone lactylation (Kla). Kla is a novel post-translational modification that occurs on lysine residues. In 2019, Zhang et al. were the first to systematically identify multiple Kla sites in mammalian cells by employing peptide immunoprecipitation in conjunction with high-sensitivity HPLC-MS/MS technology [8]. This discovery revealed that lactate can directly alter chromatin structure, thereby modulating gene expression. Its mechanism of action exhibits both similarities to, and distinct differences from, classic histone acetylation (KAC). Histone lactylation (Kla) resembles acetylation (Kac) in modifying lysine residues to loosen chromatin, yet it is uniquely driven by lactate accumulation rather than acetyl-CoA. This makes Kla a direct metabolic–epigenetic conduit through which high-lactate glioma environments activate gene programs that reinforce immunosuppression. Thus, lactylation parallels acetylation structurally but represents a distinct, tumor-linked epigenetic mechanism in glioma. Subsequent studies have revealed that lactylation occurs on a diverse array of proteins, including histones and non-histone proteins (particularly oncoproteins), and is associated with different physiological and pathological contexts [43].

The substrate donor for lactylation modification remains under active investigation. It was initially hypothesized that, analogous to the requirement of acetyl-CoA for acetylation, lactylation might require lactyl-CoA as its direct substrate. Subsequently, the presence of lactyl-CoA in mammalian cells and tissues was confirmed by liquid chromatography-tandem mass spectrometry [44]. However, the enzymatic source of lactyl-CoA remains controversial. A 2025 study reported that the mitochondrial alanyl-tRNA synthetase ACSS2 functions as a lactyl-CoA synthetase in brain tumors. It acts in concert with KAT2A to regulate histone lactylation and immune evasion [45]. Notably, the function of ACSS2 as a lactyltransferase was documented [46]. In this proposed mechanism, lactate is catalyzed by acyl-CoA synthetase short-chain family member 2 (ACSS2) to form lactyl-CoA. This lactyl-CoA is then transferred by histone acetyltransferases (HATs) to lysine residues on histones or non-histone proteins, thereby linking metabolic status with epigenetic regulation. Another study proposed that GTPSCS synthesizes lactyl-CoA directly within the nucleus, thereby coupling chromatin lactylation with the regulation of oncogenic transcription [47].

Analogous to acetylation, histone lactylation is a reversible process that is dynamically regulated by “writers” and “erasers”. These regulatory enzymes are not exclusively specific for Kla sites. It has been found that many known Histone Acetyltransferases (HATs) also possess lactyltransferase activity. Among these, p300 stands out as the first identified and most extensively characterized lactylation ‘writer’ in glioma [8]. Mechanistically, p300/CBP binds to lactyl-CoA and catalyzes the transfer of the lactyl moiety to histone lysine residues. In vitro enzymatic assays have demonstrated that CBX3 selectively potentiates the lactylation activity of P300 over its acetylation activity; notably, CBX3 is also currently the most well-documented lactylation ‘reader’ within the context of glioma. Subsequently, Lysine Acetyltransferase 2A (KAT2A), Gcn5-related N-acetyltransferase 13 (GNAT13), Histone acetyltransferase binding to ORC1 (HBO1), GNAT family N-acetyltransferase YiaC, TIP60, and Alanyl-tRNA synthetase 1 (AARS1) have also been identified as Kla ‘writers’ [45,48,49,50,51,52]. Among these, only P300 and KAT2A have been confirmed to function as lactyltransferases in brain tumors. However, research on these latter enzymes remains largely confined to biochemical mechanistic exploration. Their specific validation in clinical glioma samples remains to be fully elucidated, suggesting they currently serve as potential candidates for further investigation. Furthermore, AARS1 has been reported to function as a lactate sensor. It mediates global lysine lactylation in tumor cells and is capable of lactylating multiple proteins, including CDF and YAP [48,53]. In contrast to acetyltransferases, AARS1 directly utilizes lactate, rather than lactyl-CoA, as its substrate. Notably, however, none of the enzymes identified thus far have exhibited strict specificity for lactylation, making it difficult to determine the precise conditions under which this modification is efficiently catalyzed.

With respect to ‘erasers’, studies have clearly demonstrated that Class I histone deacetylases (HDAC1-3), as well as multiple Sirtuin family members (SIRT1-3, 6), all possess efficient delactylase activity [54,55,56,57].

Currently, the specific lactylation writers and erasers functioning within the glioma context remain poorly characterized. To address this, future studies could employ genome-wide CRISPR screening coupled with lactylation-specific antibody sorting or mass spectrometry readouts in glioma models. Given the shared enzymatic machinery between acetylation and lactylation, targeted knockout screens focusing on acetyltransferase and deacetylase families would be particularly effective for systematically identifying potential lactyltransferases and delactylases.

Histone lactylation is governed by an enzymatic system analogous to that of acetylation. This overlap suggests a complex interplay of competition or synergy between the two modifications. In high-lactate environments, the relative abundance of intracellular lactyl-CoA versus acetyl-CoA may bias the substrate selection of p300/CBP, thereby reshaping the epigenetic landscape at gene promoters and enhancers. Although p300 exhibits significantly higher catalytic efficiency (kinetics) for acetyl-CoA than for lactyl-CoA, cells can override this preference through the law of mass action—driven by metabolite concentrations—and specific inductive contexts. This shift forces p300 toward lactylation, endowing it with unique signaling functions. The relatively lower catalytic efficiency implies that lactylation serves as a specific sensor of high-lactate states rather than a constitutive housekeeping modification. Furthermore, lactate itself has been reported to directly inhibit HDAC activity, potentially leading to globally elevated levels of both acetylation and lactylation, which cooperatively promote chromatin accessibility and gene transcription. Thus, while acetylation functions as a ubiquitous, rapid-response modification reflecting real-time acetyl-CoA availability, lactylation necessitates prolonged metabolic remodeling, a high-lactate milieu, and specific synthetase-transferase complexes. Consequently, lactylation is characterized by slower kinetics, greater site-specificity, and a functional bias toward immune regulation and cellular ‘state remodeling.’ Far from being a non-specific byproduct, lactylation represents a finely gated, signal-bearing metabolic–epigenetic bridge.

### 2.3. Functional Consequences of Lactylation in Glioma

Histone lactylation, which is driven by lactate and dynamically regulated by the aforementioned enzymatic systems, translates directly into the reprogramming of key gene expression programs in glioma cells, thereby driving their malignant phenotype. In glioma models, it has been shown that high lactate levels drive histone lactylation, which directly modulates the gene expression programs of both tumor cells and immune cells, consequently promoting robust tumor progression. This process has been implicated in multiple aspects of malignancy, including immune escape, therapeutic resistance, and the maintenance of tumor stemness.

First, with respect to immune escape, lactate-driven Kla modification has been identified as a key event in establishing an immunosuppressive microenvironment. It has been demonstrated that lactate impacts not only the tumor cells themselves but also the immune cells within the microenvironment. In the GBM microenvironment, monocyte-derived macrophages (MDMs) are the dominant cell type in late-stage disease. Their high glycolytic state directs glucose flux to drive histone lactylation, which subsequently promotes IL-10 expression. This, in turn, enhances immunosuppressive function and inhibits T-cell activity. This evidence suggests that lactate-driven histone lactylation is a key event by which MDMs acquire an inhibitory phenotype [58]. In addition, hypoxia has been shown to mediate the uptake of glioma-derived lactate by macrophages via MCT1. This process leads to the upregulation of histone H3K18 lactylation and, in a histone-lactate-dependent manner, promotes the expression of Tumor Necrosis Factor Superfamily Member 9 (TNFSF9), thereby facilitating malignant GBM proliferation [59]. Lactate not only impacts myeloid cells but can also induce CBX3-dependent histone lactylation within tumor cells. This induction activates an immunosuppressive transcriptional program and upregulates CD47, thereby impairing phagocytosis [9].

Second, with respect to therapeutic resistance, lactylation modification has been shown to play a core role in the mechanisms by which glioma resists the standard chemotherapeutic agent temozolomide (TMZ). In GBM overexpressing ALDH1A3, lactate accumulation resulting from glycolytic reprogramming has been found to lactylate the DNA repair protein XRCC1. This modification enhances nuclear translocation and augments DNA damage repair capabilities, thereby conferring treatment resistance [60]. In recurrent GBM tissues and TMZ-resistant cells, histone lactylation levels have been observed to be significantly upregulated, particularly at H3K9la. Mechanistically, H3K9la is reported to confer resistance to TMZ in GBM cells by modulating LUC7L2-mediated intron splicing of the MLH1 gene [61].

Finally, with respect to the maintenance of tumor stemness, lactylation has been identified as a key regulatory mechanism driving glioma stem cell (GSC) self-renewal and proliferation. Global lactylation levels in GSCs have been found to be significantly elevated in comparison to differentiated glioma cells. For example, the lactylation of the RNA-binding protein PTBP1 has been shown to be crucial for the maintenance of GSC identity [62]. Furthermore, lactate has been reported to promote H3K18la at the VRK1 promoter region, which leads to the upregulation of VRK1 expression. This, in turn, modulates GSC stemness and proliferation via the YBX1/SOX2 pathway [63]. Hypoxia-induced overexpression of HIF-1α has been shown to upregulate YTHDF2 via H3K18la modification. This enhances the YTHDF2-BNIP3 interaction and modulates BNIP3-dependent, mitophagy-mediated metabolic reprogramming, consequently impacting glioma stemness and invasion [64].

These lactate-driven epigenetic events collectively activate a series of gene programs associated with stemness, proliferation, and survival. This action sustains the GSC population, a cell cohort that is a key driver of tumor recurrence and therapeutic failure. These findings collectively depict a clear scenario wherein lactate, acting as a nexus between metabolism and epigenetics, systematically activates multiple core programs that drive malignant glioma progression via Kla modification, thereby establishing a self-reinforcing oncogenic positive feedback loop. In the classical landscape of histone modifications, compared to rapid and broad-spectrum K-acetylation, K-lactylation requires high lactate levels and specific enzyme complexes. It exhibits slower kinetics and a more restricted site specificity, tending to encode ‘long-term metabolic and microenvironmental states.’ Conversely, modifications on arginine (R) residues remain dominated by methylation and citrullination, with no reliable evidence currently supporting the existence of histone R-lactylation. Therefore, in the context of glioma, lactylation is best characterized not as a simple subsidiary of acetylation or arginine modifications, but as a distinct, Warburg-driven signaling modification that selectively targets key lysine sites.

## 3. Lactate Shapes the Immunoepigenetic Landscape of the Glioma Microenvironment

In the preceding chapter, the impact of lactate-driven epigenetic reprogramming on the intrinsic malignant phenotypes of glioma cells (such as stemness and therapeutic resistance) was the primary focus. However, the implications of this regulatory axis extend considerably beyond these cell-intrinsic effects. The substantial quantities of lactate, which are actively effluxed by tumor cells into the microenvironment via MCTs, function as key extracellular signaling molecules and metabolites. Consequently, the ‘immuno-epigenome’ of the entire TME is being profoundly reshaped.

**Figure 2:**
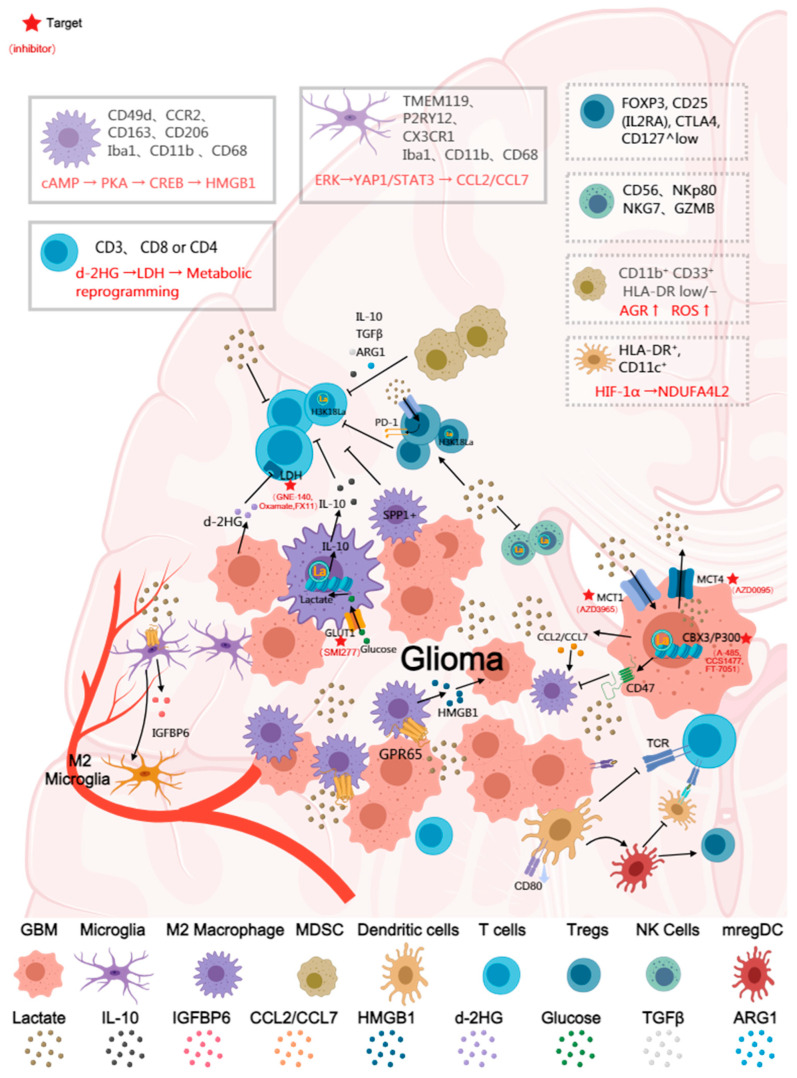
Schematic illustration of the immunosuppressive effects of lactate in the glioma tumor microenvironment. The figure depicts major immune cell types, including macrophages, microglia, T cells, regulatory T cells (Tregs), dendritic cells (DCs), natural killer (NK) cells, and myeloid-derived suppressor cells (MDSCs). Boxes summarize representative cell markers and key signaling pathways regulated by lactate (e.g., HIF-1α, STAT3). Major receptors and effector molecules are labeled in the figure, and arrows indicate the principal stimulatory and inhibitory effects of lactate on immune cells. Cells shown in dashed boxes (such as certain NK and DC subsets) represent populations for which direct evidence of lactate regulation in glioma remains limited, suggesting directions for future investigation. The red asterisks indicate drug targets.

The immune infiltrate in diffuse glioma is highly heterogeneous, and the immune landscape of the glioma tumor microenvironment is predominantly composed of myeloid cells, with lymphocytes being comparatively sparse, including macrophages, microglia, myeloid-derived suppressor cells, lymphocytes (such as CD8^+^ cytotoxic T cells, CD4^+^ regulatory T cells, and B cells), natural killer (NK) cells, and neutrophils. The dominant population is composed of resident microglia and bone marrow-derived macrophages, which are collectively referred to as glioma-associated macrophages/microglia (GAM). The immunosuppressive functions of these cells are why glioma has long been considered an immunologically ‘cold’ tumor, and this immunosuppressive state is frequently correlated with aberrant tumor metabolism [65,66,67]. Tumor-derived lactate promotes chemokine-mediated recruitment and polarization of immunosuppressive populations (TAMs, MDSCs, Tregs), thereby increasing suppressive cell infiltration into the tumor. Extensive research has indicated that lactate functions as a critical node connecting metabolism and immunity. Lactate suppresses immune function not only through extracellular acidification but also by acting as a signaling metabolite and an epigenetic substrate that directly shapes immune cell fate. Lactate promotes the immunosuppressive polarization of glioma-associated macrophages (GAMs), impairs antigen presentation by dendritic cells (DCs), induces the expansion of regulatory T cells (Tregs), and suppresses the effector activity of CD8^+^ T cells and NK cells. In doing so, it shapes an immune-evasive microenvironment on multiple levels. While lactate has been widely characterized as a central mediator of immunosuppression in gliomas, a smaller but growing body of evidence indicates that tumor-associated extracellular acidification may also modulate immune cell function. Preliminary studies suggest that low pH can diminish T-cell and NK-cell effector activity, alter antigen-presentation capacity, and bias myeloid cells toward regulatory or suppressive states [59,68]. These findings collectively imply that acidosis may represent an additional, yet underexplored, layer of metabolic regulation shaping the glioma immune microenvironment (Figure 2). Nevertheless, current data specific to glioma remain limited, fragmented, and often inferred from other tumor systems, highlighting the need for spatially resolved pH profiling, pH-responsive multi-omics analyses, and mechanistic studies in physiologically relevant glioma models. 

### 3.1. Glioma-Associated Macrophages/Microglia (GAM)

Glioma-associated macrophages/microglia (GAM) represent the most abundant immune cell population within the glioma immune microenvironment, potentially constituting 30–50% of the total tumor volume [69]. GAM comprise both brain-resident microglia and peripheral Bone Marrow-Derived Macrophages (BMDMs). Microglia are predominantly distributed at the tumor margin, whereas BMDMs are primarily localized to the tumor core, necrotic zones, and perivascular spaces, where they play a key immune-regulatory role in tumor progression [70]. Microglia are characterized by the specific expression of markers such as TMEM119, P2RY12, and CX3CR1, while peripheral macrophages exhibit high expression of molecules including CD49d, CCR2, CD163, and CD206. Both populations express common myeloid cell markers, including Iba1, CD11b, and CD68.

Given the high dependence of glioma cells on glycolytic metabolism, a substantial accumulation of lactate is observed in the tumor microenvironment. This lactate functions as an important metabolic signal that modulates GAM phenotype and function. Studies have demonstrated that LDHA and MCT1 are overexpressed in glioma and are positively correlated with the infiltration of CD163^+^/CD206^+^ M2-type GAM. It has been reported that tumor-derived lactate is sensed by the acid receptor GPR65 on GAM, which in turn induces HMGB1 secretion via the cAMP → PKA → CREB signaling pathway. This HMGB1 secretion further promotes the proliferation, migration, invasion, and mesenchymal transition of glioma cells. This interaction establishes a “lactate–macrophage–tumor cell” positive feedback loop, enhancing the immunosuppressive and pro-tumoral activities of GAM [71]. Another study identified and validated an LDHA-driven metabolic symbiosis in GBM. In this model, tumor LDHA was shown to upregulate CCL2/CCL7 via the ERK → YAP1/STAT3 pathway, thereby recruiting macrophages. In turn, these macrophages were found to enhance tumor glycolysis by secreting LDHA-containing exosomes. Genetic or pharmacological inhibition of this axis was reported to significantly inhibit tumor growth and reduce macrophage infiltration [28].

Lactate has also been shown to regulate the polarization of glioma microglia by upregulating IGFBP6 expression. Specifically, lactate induces microglia to express IGFBP6, which promotes a metabolic shift from glycolysis toward oxidative phosphorylation. This shift is accompanied by the upregulation of M2 markers (e.g., ARG1, CD206, CD163, TGF-β) and the downregulation of the M1 marker iNOS. Furthermore, IGFBP6 has been found to, in turn, enhance the migration and colony-forming ability of glioma cells [11].

The function of GAMs in glioma is also profoundly influenced by lactylation modification. It has been shown that in GBM, where BMDMs are the dominant population in late-stage disease, lactate promotes IL-10 expression via histone lactylation, thereby enhancing the immunosuppressive functions of these cells; correspondingly, the inhibition of glycolysis or lactate production was shown to reduce IL-10 levels and restore T-cell activity. GLUT1 upregulation via the PERK-ATF4 pathway was identified as a key driver of this process [58]. Furthermore, hypoxic-adapted tumor cells in brain tumors have been reported to induce histone lactylation via lactate secretion. This induction causes tumor-infiltrating macrophages to differentiate into an SPP1^+^ subtype. This macrophage subtype, not only promotes tumor cell growth via the MAPK signaling pathway but also inhibits the cytotoxic activity of CD8^+^ T cells [72]. Lactate produced by both patient-derived GSCs and microglia/macrophages has been shown to induce elevated levels of histone lactylation within GSCs and the tumor cell nucleus. The binding of CBX3 to EP300 reportedly biases EP300 toward utilizing lactyl-CoA, thereby increasing histone lactylation. This lactate-activated epigenetic reprogramming subsequently leads to CD47 upregulation, which in turn inhibits microglia/macrophage phagocytosis and supports tumor cell immune escape via pathways such as STAT3. Inhibition of CBX3 or lactate production has been found to reduce CD47 expression, restore phagocytic function, and inhibit tumor growth [9].

In summary, the primary effects of lactate on GAM in glioma can be summarized as follows: (1) Promotion of M2 phenotype polarization: The high-lactate, hypoxic environment induces GAM polarization towards an M2 phenotype, a shift that is accompanied by the upregulation of markers such as CD206, ARG1, SPP1, and TGF-β. (2) Modulation of epigenetic reprogramming: Lactate, acting via GPR65 signaling and histone lactylation, promotes the activation of immunosuppressive pathways involving IGFBP6, SPP1, and HMGB1. (3) Suppression of anti-tumor immunity: The resultant M2-type GAMs subsequently inhibit CD8^+^ T-cell function, promote angiogenesis, and drive tumor progression. Lactate, through the dual mechanisms of metabolic regulation and lactylation, drives the polarization of GAM towards an immunosuppressive phenotype. However, therapeutic strategies for depleting TAMs have not been well translated into the clinic, suggesting that the need for a deeper understanding of how lactate shapes TAM phenotype and function—insights that may reveal more effective therapeutic avenues for glioma.

### 3.2. Myeloid-Derived Suppressor Cells (MDSCs)

In glioma, myeloid-derived suppressor cells (MDSCs) represent an important immunosuppressive cell population that, despite constituting only a minority (4–8%) of CD45^+^ cells in GBM. Recent studies have delineated multiple MDSC subsets, including monocytic (M-MDSC), polymorphonuclear (PMN-MDSC), and early progenitor-like populations (E-MDSC), each exhibiting distinct transcriptional signatures and spatial localization within GBM [73]. Human M-MDSCs are typically defined as CD11b^+^CD33^+^HLA-DR^low/−^CD14^+^, whereas PMN-MDSCs express CD11b^+^CD33^dim^HLA-DR^−^CD15^+^ or CD66b^+^. MDSCs are known to play a core role in facilitating immune evasion and promoting tumor progression, angiogenesis, invasion, and metastasis. MDSCs are frequently observed to accumulate in TME, particularly in high-grade gliomas. This accumulation is detectable both within the tumor tissue (specifically in the tumor core, necrotic zones, and perivascular-rich areas) and as elevated levels in the peripheral blood of patients, and is often correlated with tumor progression and immunosuppression [74,75] MDSCs are known to inhibit T-cell activity and promote tumor progression through the secretion of immunosuppressive factors, including IL-10, TGF-β, and ARG1 [76]. Lactate, functioning as an oncometabolite, not only promotes MDSC recruitment via the acidic microenvironment but also augments their immunosuppressive function through metabolic and epigenetic mechanisms. These mechanisms include the induction of ARG1 upregulation and ROS generation, which strengthens the suppression of effector T cells. Lactate has been shown to stabilize HIF-1α and promote a GBM myeloid inhibitory phenotype. Reducing lactate levels or inhibiting lactate production has been shown to attenuate MDSC accumulation and function, suggesting that lactate metabolism is a critical upstream regulator of MDSC-mediated immunosuppression [77]. In summary, the promotion of MDSC infiltration and inhibitory activity by lactate in the glioma microenvironment represents an important contributor to tumor immune escape. Despite these advances, the precise molecular mechanisms through which lactate modulates distinct MDSC subsets in glioma—particularly regarding epigenetic programs, lactylation, and metabolic–immune crosstalk—remain incompletely understood. A deeper investigation into this lactate–MDSC axis will be essential for developing more effective strategies to reprogram or therapeutically target MDSCs in glioma.

### 3.3. Dendritic Cells (DCs)

In glioma, although dendritic cells (DCs) are crucial for recognizing and presenting tumor antigens, they bridge the innate and adaptive immune systems, but their infiltration levels are typically low and their function is often suppressed. This state reflects the inhibition of antigen presentation mechanisms by the tumor microenvironment. In recent years, it has been discovered that in activated DCs, lactate, in a HIF-1α-dependent manner, mediates the upregulation of NDUFA4L2 expression. This, in turn, inhibits mitochondrial reactive oxygen species (mtROS) production and limits the activation of the XBP1-driven inflammatory transcriptional module. This pathway constitutes a negative feedback regulatory loop that serves to prevent DC over-activation, consequently suppressing antigen presentation [78]. Tumor-derived lactate has also been shown to activate the Sterol Regulatory Element-Binding Protein 2 (SREBP2) signaling pathway in DCs, driving the transformation of conventional DCs (cDCs) into CD63^+^ mature regulatory DCs (mregDCs). This mregDC subtype is capable of migrating to tumor-draining lymph nodes, where it inhibits the antigen cross-presentation capabilities of other DCs and promotes the differentiation of Th2 and Treg cells, consequently fostering an immune-tolerant microenvironment. Inhibition of SREBP2 expression in DCs has been found to significantly enhance CD8^+^ T-cell activation and inhibit tumor progression [79]. Lactate, which is produced in high quantities via tumor cell glycolysis, not only lowers the pH of the tumor milieu but also interferes with the differentiation, maturation, and activation capabilities of DCs. For example, lactate has been reported to inhibit IL-12 p40 expression in DCs and reduce their expression of co-stimulatory molecules (such as CD80), thereby promoting a shift toward a tolerogenic phenotype [80]. Although Research on lactylation have been conducted outside the glioma context, the mechanistic principles—particularly lactate-driven H3K18la-associated transcriptional shifts—provide a compelling conceptual framework for understanding long-term DC reprogramming in glioblastoma. Although the existing research on lactate-mediated DC suppression is derived primarily from non-glioma models, it can be reasonably inferred that similar metabolic regulatory mechanisms also exist in glioma, given its characteristic high-lactate, low-pH microenvironment. These mechanisms likely synergistically impair the antigen presentation and activation functions of DCs, thereby promoting immune tolerance. Therefore, a deeper elucidation of the specific impact of lactate on DC function within the glioma microenvironment represents an important and urgently needed avenue of future research.

### 3.4. T Cells (CD8^+^, CD4^+^, Treg)

Lymphocytes account for only a small subset of total cells in GBM, ranging from 5% to 10% of all CD45^+^ cells. T-cell infiltration in glioma is typically observed to be scarce and functionally impaired, exhibiting the significant characteristics of an immunologically “cold” tumor [81]. Although detectable quantities of CD8^+^ cytotoxic T cells, CD4^+^ helper T cells, and immunosuppressive Tregs are present, their distribution is largely restricted to the tumor’s infiltrative margin; they are observed to be extremely scarce within the tumor core [82]. The lactate-enriched acidic microenvironment is recognized as one of the key metabolic factors contributing to T-cell dysfunction. High lactate concentrations have been shown to inhibit T-cell glycolytic metabolism and IFN-γ production, which diminishes the cytotoxic activity of CD8^+^ T cells [83]. concurrently, lactate has been observed to promote the differentiation and survival of Foxp3^+^ Tregs, thereby exacerbating immunosuppression [84]. In summary, lactate remodels T-cell function via the dual mechanisms of metabolic competition and signaling regulation, subsequently impairing the anti-tumor immune response and promoting glioma immune escape.

In contrast to the previously discussed inhibitory effects of lactate, IDH-mutant gliomas exhibit another form of metabolite-mediated immunosuppression: Their oncometabolite, D-2HG, not only impacts the tumor itself but also modulates immune cell function through tumor-non-autonomous mechanisms. It has been demonstrated that D-2HG can be taken up by CD8^+^ T cells, subsequently causing rapid and reversible alterations to their metabolic state and anti-tumor activity. These alterations are manifested as suppressed glycolysis, reduced cytotoxicity, and impaired IFN-γ signaling. Mechanistically, D-2HG is reported to target LDH, driving T cells into a metabolically reprogrammed state and consequently impairing their anti-tumor function. This effect was also validated in clinical samples from IDH1-mutant glioma patients, indicating that oncometabolites can promote immune escape by directly modulating T-cell metabolism and function [85]. Research employing large-scale RNA-seq and single-cell analyses has found that high lactate levels promote the formation of an immunosuppressive microenvironment in glioblastoma, which significantly inhibits CD8^+^ T-cell migration and tumor infiltration. This effect was subsequently validated using co-culture and immunohistochemistry [12]. A similar lactate-driven lactylation mechanism likewise operates in the glioma microenvironment, shaping T-cell responses. A study on glioma CAR-T therapy revealed that lactate induces H3K18 lactylation in CD4^+^ T cells and Tregs. This induction upregulates CD39/CD73/CCR8 expression, thereby reinforcing the immunosuppressive phenotype of the glioma. Conversely, blocking lactate production was shown to significantly restore CAR-T cell function [12].

With respect to the promotive effect of lactate on Treg cells, it has been found that in low-glucose environments, Treg cells actively take up lactate via MCT1. This process promotes NFAT1 translocation to the nucleus, subsequently enhancing PD-1 expression; in contrast, PD-1 expression in effector T cells was observed to be inhibited. This mechanism confers an immunosuppressive advantage upon Tregs within the TME, which may, in turn, limit the efficacy of PD-1 inhibition therapy [86]. Additional studies have revealed that lactate enhances Treg stability and immunosuppressive function via the lactylation of the MOESIN protein at the K72 site. Lactate has been shown to promote MOESIN interaction with TGF-β receptor I and to activate the SMAD3 signaling pathway, thereby enhancing Treg induction and function. Inhibition of lactate production or promotion of lactate degradation has been found to weaken Treg induction and enhance anti-tumor immunity. Furthermore, combining LDH inhibitors with anti-PD-1 therapy was shown to significantly improve anti-tumor efficacy [87]. Lactate has also been reported to promote USP39-mediated RNA splicing, which facilitates CTLA-4 expression in a Foxp3-dependent manner. This increase in CTLA-4 RNA splicing efficiency was observed in tumor-infiltrating Treg cells from colorectal cancer patients [88].

In summary, lactate, acting via dual metabolic and epigenetic mechanisms, synergistically suppresses the function of CD8^+^ and CD4^+^ T cells while promoting the immunosuppressive activity of Tregs. This dual action establishes a ‘metabolic–immune co-inhibitory’ microenvironment that is characteristic of glioma. Prospectively, interventions targeting the lactate metabolic pathway or lactylation signaling (e.g., LDHA inhibitors, MCT blockers, or lactylation-modulating drugs) represent a promising strategy to restore T-cell effector function, augment immunotherapy responses, and provide new strategic directions for glioma immunotherapy. Although direct research elucidating lactate’s regulation of Treg cell function in glioma is currently lacking, evidence from other solid tumors suggests that lactate promotes Treg stability and immunosuppressive activity. Future work should integrate multi-omics profiling, spatial metabolism, and epigenetic analysis to delineate lactate-responsive pathways, clarify the contribution of Kla to T-cell exhaustion, and identify subtype-specific vulnerabilities in IDH-wild-type gliomas. Such insights may ultimately enable the development of targeted metabolic or epigenetic strategies to restore anti-tumor T-cell immunity and enhance the efficacy of immunotherapies in glioma.

### 3.5. NK Cell

Natural killer (NK) cells are innate immune cells with strong anti-tumor activity and may offer a promising treatment strategy for GBM. Tumor-derived lactate and the accompanying microenvironment acidification are known to inhibit NK cell effector functions (e.g., the release of perforin, granzyme B, and IFN-γ), downregulate activating receptor expression, and impair cytotoxicity against tumor cells [59,89]. Recent studies have indicated that lactate can impair the anti-tumor function of NK cells by inducing intracellular Kla. In high-lactate environments, NK cells exhibit increased lactylation levels, impaired NAD^+^ metabolism, mitochondrial fragmentation, and decreased cytotoxicity. It has been demonstrated that supplementation with the NAD^+^ precursor nicotinamide riboside, in combination with the SIRT3-activating compound honokiol, can effectively reduce intracellular lactylation levels, thereby restoring NK cell mitochondrial homeostasis and cytotoxic activity. Mechanistically, this combination therapy was shown to modulate the lactylation state of ROCK1 and inhibit the ROCK1–DRP1 signaling pathway, thereby preventing mitochondrial fragmentation and enhancing the anti-tumor efficacy of NK cells [90]. This study was the first to reveal the key role of lactylation in NK cell dysfunction. Early in vivo and in vitro studies have observed similar effects in multiple tumor models, indicating that lactate is an important metabolic factor in the suppression of tumor immune surveillance. Recent work demonstrates that NK-cell activity in GBM is strongly shaped by epigenetic programs, as exemplified by IL-21–engineered NK cells, whose enhanced anti-tumor function is driven by C/EBP-dependent chromatin remodeling [91]. Given emerging evidence that lactate-induced lysine lactylation can similarly reprogram NK-cell chromatin and impair cytotoxicity, it is highly likely that GBM-associated lactate accumulation contributes to NK-cell dysfunction through analogous epigenetic mechanisms. Defining this lactate–lactylation axis in GBM-infiltrating NK cells represents an important and promising direction for future research.

## 4. Integration of Metabolic and Immune Epigenetic Reprogramming in Glioma Progression

### 4.1. The Lactate–Epigenetic–Immune Feedback Loop

A complex interplay exists among lactate, epigenetic modifications, and the immune response within the glioma microenvironment. Lactate accumulation is not merely a consequence of tumor metabolism; it also functions as a signaling molecule capable of influencing immune cell function via the modulation of epigenetic mechanisms. Specifically, high tumor-derived lactate concentrations are capable of inducing epigenetic alterations, promoting the expression of immunosuppressive factors, and consequently impairing the anti-tumor activity of immune cells. In turn, immune cells that are modulated by epigenetic modifications can also impact tumor cell metabolism, further promoting lactate production and accumulation and thereby establishing a self-reinforcing vicious cycle. Therefore, a thorough understanding of the dynamic equilibrium among lactate, epigenetics, and immunity is essential for elucidating the immune escape mechanisms of glioma and for developing more effective immunotherapy strategies.

Glycolytic metabolites, particularly lactate, have been shown to exert a dual role in immune cells: they can support the function of activated immune cells, yet upon accumulation, they can also inhibit immune cell function via signal transduction and epigenetic modifications, thereby influencing the progression of both tumors and chronic inflammatory diseases.

### 4.2. Spatial and Single-Cell Omics Evidence

Single-cell sequencing technologies (e.g., scRNA-seq and scATAC-seq) have been widely employed in the study of the tumor microenvironment, revealing the immunosuppressive characteristics of high-lactate regions. These studies have primarily focused on three areas: first, the construction of cellular atlases of the tumor microenvironment has revealed that high-lactate regions are enriched with immunosuppressive cell types, including M2 macrophages, MDSCs, and Tregs; second, the analysis of immune cell functional states has demonstrated that immune cells in high-lactate regions exhibit high expression of immune checkpoint molecules, secrete immunosuppressive cytokines, and display metabolic features such as enhanced glycolysis; third, the investigation of chromatin accessibility has elucidated the epigenetic regulatory mechanisms underlying immune suppression-related genes, such as reduced chromatin accessibility and diminished transcription factor binding. These findings collectively elucidate the complex immunosuppressive regulatory network within the high-lactate microenvironment.

An integrative analysis of lung adenocarcinoma (LUAD) employing single-cell transcriptomic sequencing (scRNA-seq) has revealed that lactate metabolism plays a crucial role in shaping the tumor immune microenvironment. Lactate metabolic activity was evaluated at the single-cell level using the AUCell algorithm. This analysis led to the identification of 590 lactate metabolism-related genes (LMRGs) and the subsequent construction of a prognostic model based on five of these key genes. The results indicated that cell populations with high lactate metabolic activity were closely correlated with immunosuppressive characteristics. These were manifested as reduced infiltration of CD8^+^ cytotoxic T cells and NK cells, a significant increase in Tregs and M2-type macrophages, and a concurrent upregulation of immune checkpoint molecules such as PD-L1 and CTLA-4. These results reveal that enhanced lactate metabolism drives immune exhaustion and immune escape, indicating that targeting lactate metabolism may represent a promising strategy to improve the response of LUAD patients to immunotherapy [92]. Furthermore, another study, which combined single-cell transcriptomics with bulk RNA-sequencing analysis, revealed that lactate significantly shapes the immunosuppressive microenvironment in GBM. The study classified over 1400 GBM samples and performed validation using the Xiangya cohort. It was found that tumors with high lactate levels exhibited characteristics such as reduced CD8^+^ T-cell infiltration and the upregulation of immune checkpoint genes. Further cellular interaction analyses and co-culture experiments indicated that lactate inhibits the migration and activation of CD8^+^ T cells, thereby promoting immune escape. This study elucidated the key role of lactate metabolism in the establishment of GBM immunosuppression and provided potential directions for metabolically targeted immunotherapy [12].

Multi-omics studies have revealed that lactate exerts broad epigenetic regulatory effects during tumor progression via protein lactylation. A combined proteomic, transcriptomic, and chromatin accessibility analysis of oral squamous cell carcinoma (OSCC) has demonstrated that lactylation modifications are widely distributed on both metabolism- and transcription-related proteins. These modifications were also shown to significantly alter chromatin structure and gene expression patterns. Lactate treatment was observed to upregulate the expression of various immune and metabolic regulatory genes. Notably, lactylation at a DHX9 site was confirmed to release its tumor-suppressive constraints, thereby promoting tumor cell proliferation and migration [93].

Multi-omics studies have confirmed that tumor immune infiltration is influenced by lactate via metabolic reprogramming and epigenetic regulation. This influence is manifested as decreased CD8^+^ T-cell activity and an enrichment of immunosuppressive cells. Although research specific to glioma remains relatively limited, findings from other tumor types suggest that lactate also plays an important role in establishing the immunosuppressive microenvironment in glioma, and it is therefore hypothesized that lactate may represent a key target for future metabolic–immune combination therapies.

Although multi-omics studies increasingly implicate lactate metabolism in shaping the immunosuppressive landscape of glioma, current evidence remains largely associative, with limited mechanistic insight into how lactate-driven metabolic, signaling, and epigenetic programs converge to rewire immune cell function. Key gaps include the absence of spatially resolved datasets linking lactate abundance to cell-type–specific chromatin states, insufficient characterization of lactate-derived histone and non-histone lactylation in glioma-infiltrating immune subsets, and a lack of functional perturbation studies establishing causality. Moving forward, integrating spatial metabolomics with single-cell transcriptomic and epigenomic profiling, alongside isotope tracing and targeted modulation of lactate transporters or lactylation machinery, will be essential for defining lactate–immune interactions with precision. Such mechanistic clarity will not only refine our understanding of metabolic–epigenetic–immune crosstalk but also support the rational development of therapies such as LDHA or MCT inhibition in combination with immunotherapy—particularly in lactate-high, immune-cold glioma subtypes.

### 4.3. Clinical Correlation

Glioma, a highly invasive and therapeutically challenging brain tumor, is characterized by the dysregulation of its immune microenvironment, which is understood to play a critical role in tumor progression and resistance. It has been demonstrated that glioma cells undergo metabolic reprogramming, resulting in the substantial production and accumulation of lactate within the local tumor milieu. This accumulated lactate not only promotes the recruitment and activation of immunosuppressive cells but also suppresses the anti-tumor functions of potent immune effector cells, including CD8^+^ T cells and NK cells, thereby creating an immune-evasive “niche”. This mechanism significantly impairs the anti-tumor capacity of the immune system. It is considered a core factor driving glioma immunoresistance.

To address the role of lactate in immunosuppression, various therapeutic strategies are being progressively developed. First, agents targeting the lactate metabolic pathway, such as LDH inhibitors, are designed to reduce lactate production and modulate the inflammatory status of the immune microenvironment. Second, immunotherapies, particularly immune checkpoint inhibitors, aim to reverse the immunosuppressive state of T cells and augment the anti-tumor immune response. Furthermore, the use of antiviral drugs, immunomodulators, or nanocarriers is also being explored to optimize the delivery of lactate metabolism inhibitors to the tumor microenvironment, thereby enhancing therapeutic efficacy.

In recent years, combination strategies have garnered significant attention. These approaches typically involve augmenting immune checkpoint inhibition with lactate metabolism inhibitors in an effort to reverse lactate-induced immunosuppression. Furthermore, immune cell therapies are also being investigated in combination with lactate modulation strategies to enhance their anti-tumor activity. Prospectively, personalized metabolic–immune regulatory regimens are poised to become an important avenue for improving prognoses in glioma patients. This series of innovative therapeutic approaches, based on a thorough understanding of the role of lactate in the tumor immune microenvironment, offers new hope for the clinical management of glioma.

## 5. Therapeutic Targeting of the Lactate–Epigenetic–Immune Axis

Targeting lactate metabolism offers a promising avenue to disrupt the metabolic–epigenetic–immune circuitry that fuels glioma progression. Inhibitors of LDHA, MCT1/4, or GPR81 can reduce lactate accumulation and restore immune function, while epigenetic modulators such as p300 and HDAC inhibitors may rebalance aberrant histone lactylation and acetylation states. Combining metabolic inhibitors with immune checkpoint blockade has shown synergistic effects in preclinical glioma models, underscoring the potential of integrative immunometabolic therapy. However, challenges remain, including blood–brain barrier penetration, metabolic heterogeneity, and incomplete knowledge of lactylation-specific enzymes, which must be addressed to translate these findings into clinical benefit (Table 1). 

### 5.1. Targeting Lactate Metabolism

In IDHmut gliomas, the lactate metabolic chain—encompassing production, efflux, and reuse—exhibits a general downregulation. Consequently, lactate production is reduced, MCT1/MCT4-mediated transmembrane flux is diminished, and a comparatively greater dependence on lactate reuse and mitochondrial oxidative phosphorylation (OXPHOS) is observed. As a result, the contribution of “lactate-driven epigenetic-immune reprogramming” is correspondingly diminished. In contrast, biological features mediated by the oncometabolite D-2-HG—such as epigenetic remodeling and immune modulation—as well as the redox vulnerability stemming from NADPH consumption, emerge as the dominant characteristics of this tumor subtype. These differences not only delineate a stratified profile of IDHmut versus IDHwt gliomas along the metabolic–epigenetic–immune axis but also indicate that distinct therapeutic priorities and combinatorial strategies are required for each. Therefore, therapeutic strategies targeting the lactate-dominated pathogenic pathway will be the exclusive focus of this review.

Agents targeting the glycolytic pathway or lactate transport are currently under preclinical investigation as a strategy to counter the Warburg effect and its immunosuppressive consequences. GLUT1 and GLUT3 are primarily expressed during the glycolytic process. Although targeting histone Kla with GLUT1 inhibitors has shown promise, this approach may also impact other glucose-related processes. LDH inhibitors are designed to prevent the conversion of pyruvate to lactate, while MCT inhibitors are intended to block the transmembrane transport of lactate.

The small molecule inhibitor SMI277 has been identified as a promising lead compound for the development of GLUT1-specific cancer therapeutics. It is reported to bind to amino acid residues Q283, F379, and E380 within the GLUT1 glucose transport channel. This binding subsequently results in a dose-dependent reduction in lactate levels [94]. Ex vivo experiments have demonstrated that SMI277 is capable of enhancing the CD8^+^ T-cell response.

LDH inhibitors (LDHis) represent a class of small-molecule compounds designed to specifically target and inhibit the catalytic activity of Lactate Dehydrogenase A (LDHA). LDHA functions as a critical rate-limiting enzyme at the terminal step of glycolysis, where it catalyzes the reduction of pyruvate to lactate coupled with the oxidation of NADH to NAD^+^. The small molecule inhibitor FX11, a representative LDH inhibitor (LDHi), is a classic agent used for in vivo and in vitro mechanistic validation. In multiple models, it has been demonstrated to inhibit aerobic glycolysis, induce oxidative stress, and exert tumor-suppressive effects [95,96]. However, its clinical translation has been limited by poor pharmacokinetics and selectivity, restricting its use primarily to mechanistic studies and combination strategies. GNE-140, another LDHi, has been shown in preclinical models to redistribute intratumoral glucose, inhibit tumor cell glycolysis, and induce solid tumor regression [97,98]. Despite these findings, these inhibitors have not yet entered clinical trials. Oxamate, a classic LDH inhibitor, competitively inhibits LDH by mimicking pyruvate. It is predominantly used in in vitro experiments, though it has also been employed in some animal models; it has not, however, advanced to clinical use [99,100].

MCT inhibitors (MCTi) constitute a category of compounds targeting the monocarboxylate transporter (MCT) family, with a particular focus on MCT1 and MCT4. Biologically, MCTs operate as proton-linked symporters that facilitate the transmembrane flux of monocarboxylates, including lactate, pyruvate, and ketone bodies. Numerous small compounds exhibiting significant inhibitory activity against MCT1 and MCT4 have been investigated in preclinical studies. Among these, the inhibitor AZD3965 has advanced to Phase I clinical trials [101]. AZD3965, a selective inhibitor of MCT1, is designed to impede lactate efflux from cancer cells. This effect is particularly pronounced in malignancies with reduced or absent MCT4 expression, resulting in intracellular acidification and metabolic dysregulation. In GBM cell lines, such as U87MG and A172, the inhibition of MCT1 by AZD3965 has been shown to inhibit proliferation [33]. AZD0095, a selective MCT4 inhibitor, is designed to inhibit tumor lactate efflux and reverse lactate-driven immunosuppression [102]. Currently, no publicly available data from human or animal glioma models evaluating AZD0095 have been reported.

### 5.2. Epigenetic Modulators

p300/CBP inhibitors (p300i) are a class of small-molecule inhibitors that specifically target the catalytic core (HAT domain) of E1A-binding protein p300 and its homolog CBP. Mechanistically, they directly inhibit the transfer of the lactyl moiety from lactyl-CoA to histone lysine residues. The p300/CBP proteins possess both a HAT catalytic domain and “reader/recruitment” modules, such as the bromodomain (BD). These modules are responsible for recognizing acetylated lysine residues and subsequently recruiting large protein complexes to chromatin. A-485 is a selective inhibitor of the p300/CBP HAT catalytic domain that competes with the Ac-CoA binding site, thereby directly suppressing its “writer” activity [103,104]. In cellular and mouse models, it has been shown that A-485 induces the rapid and broad downregulation of active markers, including H3K27ac. This agent also inhibits super-enhancer-driven oncogenic networks, resulting in the marked suppression of tumor proliferation and oncogenic transcriptional programs in models such as prostate cancer. Furthermore, A-485 has also been identified as a novel compound capable of inhibiting inflammatory cytokine expression by macrophages and alleviating liver injury.

CCS1477 (inobrodib) is a selective P300/CBP bromodomain inhibitor. This agent blocks the “reader-recruitment” function of p300/CBP, which results in the eviction of these proteins from a subset of highly acetylated enhancers [105]. This, in turn, leads to the downregulation of downstream oncogenic networks and the induction of differentiation. In Phase I exploratory studies in prostate cancer and hematologic malignancies, CCS1477 has demonstrated preliminary activity and an acceptable safety profile. Furthermore, as a preclinical combination for immunotherapy (with anti-PD-1/PD-L1 antibodies), enhanced transcriptional and functional signals have been observed [106,107,108]. Remarkable effects of bromodomain inhibition have been observed in medulloblastoma cell lines. In comparison to A485, a widespread loss of dependency networks was observed in Group 3 medulloblastoma following treatment with CCS1477 [115]. FT-7051 represents another EP300/CBP bromodomain inhibitor and is currently undergoing Phase I development [109]. To date, no human data concerning this agent’s application in brain tumors have been reported. Should human exposure data and information regarding brain penetration become available in the future, its evaluation in basket trials for GBM subtypes may be warranted.

Histone deacetylase inhibitors (HDACi) are a class of small molecules that function as either broad-spectrum or selective inhibitors of histone deacetylase (HDAC) activity. HDACis have been extensively studied in glioma/GBM. Their mechanism of action involves inhibiting deacetylation, which promotes chromatin accessibility and reprograms oncogenic and immune transcriptional networks. Four such agents have received FDA approval (e.g., vorinostat (SAHA), panobinostat, romidepsin, and belinostat). Although their efficacy as monotherapies is limited, they are being investigated as sensitizers for radiotherapy/chemotherapy or as components of combination immunotherapy regimens. Vorinostat (SAHA), which inhibits Class I/II HDACs, is known to generally increase histone acetylation. It is currently approved for the treatment of cutaneous T-cell lymphoma [110]. Panobinostat, a potent pan-HDAC inhibitor, has been approved for the treatment of multiple myeloma. Currently, Phase I/II studies in glioma have demonstrated that convection-enhanced delivery (CED) of panobinostat is safe and feasible. This approach enables achievable local exposure, offering a potential strategy for overcoming the BBB [112,116]. In glioma clinical research, a belinostat dose-escalation trial built upon a standard chemoradiotherapy (CRT) regimen is currently underway. This study is poised to establish a novel clinical trial paradigm. This approach involves the initial application of ‘metabolic imaging’ (e.g., MRSI) during the preoperative or treatment period to assess tumor status, followed by ‘epigenetic pharmacodynamic’ monitoring to determine if the drug is exerting its intended effect at the epigenetic level [114].

### 5.3. Immunometabolic Combination Therapy

Lactate is continuously effluxed by highly glycolytic tumors via transporters such as MCTs, resulting in lactate accumulation and acidification within the microenvironment. This acidic, high-lactate environment exerts a dual detrimental effect: it not only directly impairs key immune effector cells but also promotes the proliferation of immunosuppressive cells, such as Tregs. Concurrently, lactate is also capable of reprogramming immune cell function at the genomic level, either via lactylation modifications or through the activation of specific receptors (HCAR1/GPR81). The detrimental impact of lactate and acidification on immunity has been confirmed by numerous studies. Based on this understanding, theoretical therapeutic strategies have been proposed: It is hypothesized that by inhibiting MCT1/4 to block lactate flux, or by directly neutralizing the acidic environment, it may be possible to restore the suppressed anti-tumor immune function, thereby significantly enhancing response rates to immune checkpoint inhibitors (ICIs).

A reproducible synergistic effect resulting from the combined inhibition of lactate flux and the PD-1 axis has been observed in various tumor models. Specifically, selective inhibition of MCT4 has been shown to reduce lactate efflux and elevate intratumoral pH, which subsequently enhances leukocyte infiltration and CD8^+^ T-cell activation. When combined with immune checkpoint blockade (ICB), including anti-PD-1 therapy, this approach significantly delays tumor growth and prolongs survival [33,117]. In contrast, MCT1 inhibition alone is often compromised by the MCT4 bypass pathway, and its therapeutic benefit is consequently limited in certain contexts. Although human clinical combination data have not yet been published, the Phase I study of AZD3965 (MCT1) and the clinical candidate status of AZD0095 (MCT4) have laid the foundation for subsequent clinical translation [102,118]. For the glioma subpopulation characterized by IDH-wild-type status, high MCT4 expression, and a high lactate burden, it is recommended that the pharmacodynamic chain—wherein lactate reduction leads to increased pH and subsequent immune activation—be validated in the preoperative window. Furthermore, optimization of the dosing sequencing and delivery strategies is also warranted.

The synergistic interaction between epigenetic drugs and Immune Checkpoint Inhibitors (ICIs) represents a significant area of investigation in the field of tumor immunotherapy. The fundamental role of ICIs is to ‘release the brakes’ on the immune system. This action enables T cells, in particular, to re-engage and attack tumor cells. However, in a significant subset of tumors, effective T-cell infiltration is impeded by low immunogenicity or the presence of an immunosuppressive microenvironment. This, in turn, results in poor therapeutic efficacy or the development of resistance to ICI therapy. Epigenetic drugs, such as DNA methyltransferase inhibitors (DNMTis) and histone deacetylase inhibitors (HDACis), have been demonstrated to possess potent immunomodulatory functions. The combination of these agents with ICIs is intended to overcome ICI resistance via epigenetic reprogramming, thereby achieving a synergistic effect.

The Phase I/II trial (PNOC015) evaluating Panobinostat administered via convection-enhanced delivery (CED) to patients with pediatric diffuse midline glioma has demonstrated that this intracranial delivery method is safe, feasible, and capable of achieving effective exposure [112]. The CED route thus provides a validated paradigm for the intracranial delivery of epigenetic agents. This approach can subsequently be explored sequentially or concurrently with ICIs or radiotherapy. HDAC inhibitors have entered clinical trials for both newly diagnosed and recurrent GBM, where they are being evaluated in combination with radiotherapy, temozolomide (TMZ), anti-angiogenic agents, and proteasome inhibitors. Currently, this approach represents the most viable direction for clinical translation.

### 5.4. Challenges and Perspectives

Based on a comprehensive evaluation of clinical translational maturity and mechanistic plausibility, Class I HDACs emerge as the most viable metabolic–epigenetic intervention targets in glioma, supported by multiple human trials in combination with radiotherapy and temozolomide. Following these are MCT1 inhibitors, which have entered Phase I/II trials, and the rapidly advancing CBP/p300 inhibitors. Positioned at the critical nodes of lactate transport and acetylation/lactylation writing, respectively, these targets offer dual regulatory advantages. Conversely, while LDHA demonstrates robust antitumor potency in glioma models, the absence of formal human trials currently designates it as a medium-to-long-term ‘high-risk, high-reward’ target, requiring validation of safety and efficacy through rigorously designed early-phase exploratory studies. Despite the immense promise of targeting the ‘Metabolic–Epigenetic–Immune’ axis, translating this paradigm into effective clinical therapies faces several critical challenges:

The primary challenge in targeting glioma remains the physical barrier imposed by the blood–brain barrier (BBB). Since most therapeutic agents cannot effectively penetrate the intact BBB, the prioritization of brain-penetrant molecules is essential. Compounding this issue is the fact that BBB disruption in glioma is heterogeneous and ‘patchy,’ leading to highly uneven drug distribution. This non-uniformity allows a subset of tumor cells to survive in regions of sub-therapeutic drug concentration, thereby accelerating the emergence of drug resistance. To overcome this obstacle, researchers are exploring diverse delivery strategies, such as direct infusion via convection-enhanced delivery (CED), temporary barrier opening using focused ultrasound (FUS), or the deployment of nanocarriers. However, these technologies still require rigorous validation of their standardized pharmacokinetics/pharmacodynamics (PK/PD) and safety profiles. As a smart delivery platform endowed with dual capabilities of blood–brain barrier (BBB) penetration and tumor targeting, Ferritin-armed EVs offer a feasible and highly promising strategy for the combinatorial treatment of glioblastoma [119]. This approach stands as an exemplary paradigm in the current landscape of research into BBB-crossing nanosystems.

A second major challenge is tumor metabolic heterogeneity. Gliomas are not monolithic; rather, they are composed of heterogeneous cell subpopulations possessing different metabolic phenotypes. For instance, some cells rely heavily on high glycolysis, while others depend on OXPHOS. Furthermore, metabolic pathways are also fundamentally reshaped by the tumor’s IDH mutation status. This heterogeneity implies that the use of a single metabolic inhibitor is highly susceptible to failure. This is because tumor cells can circumvent the blockade by activating ‘bypass’ or ‘compensatory’ pathways. Therefore, future strategies must shift toward dynamic and personalized combination therapies. This approach necessitates the use of functional imaging and spatial omics technologies to assess a patient’s metabolic state in real time, thereby guiding precision dosing.

A third major challenge is the reversal of the immunosuppressive microenvironment. Gliomas are considered classic ‘cold tumors,’ which is reflected in their extremely low response rates to immune checkpoint inhibitors (ICIs). This challenge stems from a twofold problem: on the one hand, as previously discussed, the high-lactate and acidic environment impairs the function of DCs and CD8^+^ T cells; on the other hand, low intrinsic tumor immunogenicity impedes T-cell recognition. Therefore, a key objective for future research is to determine how to safely ‘pre-warm’ the TME without inducing severe neurotoxicity. Achieving this goal will necessitate sophisticated combinatorial strategies. First, acidic immunosuppression must be reversed through ‘metabolic correction’. Concurrently, ‘epigenetic priming’ agents must be employed to ‘reinvigorate’ exhausted T cells and to promote antigen presentation by tumor cells. The realization of this objective will depend on the meticulous optimization of drug dosing and administration sequencing.

## 6. Conclusions

Lactate, which was traditionally regarded as a mere end-product of glycolysis, is now recognized as a critical nexus connecting metabolic reprogramming, epigenetic modifications, and immune modulation. This review systematically elucidates how, in IDHwt glioma, the substantial accumulation of lactate driven by the Warburg effect transcends its conventional role as a metabolic waste product, transforming it into a key signaling molecule and epigenetic substrate.

Serving as a substrate for modifications such as Kla, lactate translates the cell’s highly glycolytic metabolic status into specific chromatin conformations and transcriptional programs. This process exerts a dual critical effect: on the one hand, it drives cell-intrinsic malignant phenotypes associated with stemness maintenance, proliferation, and therapeutic resistance; on the other hand, it profoundly reprograms the ‘immuno-epigenome’ of various immune cells, thereby systematically establishing an immunosuppressive niche.

Therefore, this lactate-centric ‘metabolic–epigenetic–immune’ regulatory axis, which incorporates tightly coupled positive feedback loops, provides a unified theoretical framework by which the highly aggressive nature and immune escape mechanisms of glioma can be understood. This framework not only highlights the tumor’s metabolic vulnerabilities but also presents new avenues for therapeutic intervention, such as combinatorial strategies combining targeted lactate metabolism with epigenetic modulation and immune checkpoint blockade.

Despite significant progress in our understanding of this field, many key questions remain unresolved. Although an increasing number of enzymes have been identified as Kla ‘writers’, the key enzymes responsible for lactyl-CoA synthesis, as well as the specific Kla ‘erasers’, in glioma remain incompletely understood or controversial. For instance, in the high-lactate TME, it remains unclear whether the lactate molecule itself or the concomitant microenvironment acidification plays the dominant role in suppressing immune cell function. The respective contributions and synergistic interactions of these two factors have not yet been precisely delineated. As previously discussed, much of the direct evidence concerning lactate-mediated modulation of DC and Treg function is derived from non-glioma models. Consequently, their precise mechanisms of action within the glioma in situ microenvironment require further validation. Beyond these specific questions, significant challenges—including BBB penetrability and tumor heterogeneity—remain. However, the application of single-cell multi-omics, spatial metabolomics, and advanced functional metabolic imaging technologies is poised to enable a more precise spatiotemporal resolution of the dynamic equilibrium of this regulatory network.

Targeting multiple nodes along this core axis, particularly through the rational design of combination therapies capable of reshaping the tumor metabolic and immune microenvironment, represents a promising approach. It is anticipated that this will lead to the discovery of effective precision therapeutics for glioma, a devastating disease, and ultimately improve patient outcomes. Future research must continue to thoroughly investigate the intricate details of this compelling field and focus on translating basic scientific discoveries into effective clinical practice. This endeavor holds the potential to ultimately offer new hope for glioma patients.

## Figures and Tables

**Table 1:** Mechanisms of action and clinical development status of selected agents targeting the metabolic–epigenetic axis.

Therapeutic Agent	Drug Class	Mechanism of Action	Key Clinical Trials	Development/Approval Status	References
SMI277	GLUT1 Inhibitor	Binds GLUT1 channel; reduces glucose uptake and lactate levels.	—	Preclinical	[94]
FX11	LDH Inhibitor	Inhibits aerobic glycolysis; induces oxidative stress and tumor suppression	—	Preclinical	[95,96]
GNE-140	LDH Inhibitor	Inhibits glycolysis; redistributes intratumoral glucose; induces tumor regression.	—	Preclinical	[97,98]
Oxamate	LDH Inhibitor	Pyruvate mimic; competitively inhibits LDH.	—	Preclinical	[99,100]
AZD3965	MCT Inhibitor	Selective MCT1 blockade; impedes lactate efflux, causing intracellular acidification.	NCT01791595	Phase I	[101]
AZD0095	MCT Inhibitor	Selective MCT4 blockade; inhibits lactate efflux to reverse immunosuppression.	—	Clinical Candidate	[102]
A-485	p300/CBP Inhibitor	Competes with lactyl-CoA; suppresses “writer” activity;	—	Preclinical	[103,104]
CCS1477 (inobrodib)	p300/CBP Inhibitor	Blocks “reader-recruitment” function; evicts p300/CBP from enhancers.	NCT04068597 NCT03568656	Phase I	[105,106,107,108]
FT-7051	p300/CBP Inhibitor	BloScks “reader-recruitment” function.	NCT04575766	Phase I	[109]
Vorinostat (SAHA)	HDAC Inhibitor	Inhibits deacetylation; generally increases histone acetylation.	NCT00731731	FDA-approved (CTCL)	[110,111]
Panobinostat	HDAC Inhibitor	broad inhibition of HDACs.	PBTC-047 PNOC015	FDA-approved (Multiple Myeloma)	[112,113]
Belinostat	HDAC Inhibitor	Inhibits deacetylation	NCT02137759	FDA-approved (PTCL)	[114]

## Data Availability

No new data were created or analyzed in this study.
